# Subclavian-to-internal carotid artery bypass with carotid endarterectomy for total occlusion of the common carotid artery under awake neurologic monitoring: a case series

**DOI:** 10.3389/fcvm.2026.1959220

**Published:** 2026-10-02

**Authors:** Yerbol Dogalbayev, Kuralay Ilbekova, Ruslan Tukinov, Gulnar Mustafinova, Nurlan Azimzhanov, Seilbek Beketov, Didar Kadyrbek, Tokan Sultanaliyev, Irlan Sagandykov, Alexandr Fursov

**Affiliations:** 1 Professor G. V. Tsoi Scientific and Educational Center of Surgery, NJSC “Astana Medical University”, Astana, Kazakhstan; 2 Department of Interventional Radiology, CF University Medical Center, Astana, Kazakhstan; 3 Scientific Research Institute of Radiobiology and Radiation Protection, NJSC “Astana Medical University”, Astana, Kazakhstan; 4 Clinical Academic Department of Anesthesiology and Intensive Care, CF University Medical Center, Astana, Kazakhstan; 5 Center for Vascular and Reconstructive Surgery, National Research Oncology Center, Astana, Kazakhstan

**Keywords:** awake neurologic monitoring, carotid endarterectomy, case series, regional anesthesia, subclavian-carotid bypass, total common carotid artery occlusion

## Abstract

**Background:**

Total occlusion of the common carotid artery (CCA) from its origin is rare, occurring in less than 1% of brachiocephalic stenoses. Surgical revascularization is technically demanding, especially with tandem severe ipsilateral internal carotid artery (ICA) stenosis. Procedures under regional anesthesia with continuous neurologic monitoring are rarely described.

**Objective:**

To evaluate outcomes of subclavian-to-internal carotid artery bypass with carotid endarterectomy (CEA) in symptomatic patients with total CCA occlusion and severe ipsilateral ICA stenosis under regional anesthesia with awake neurologic monitoring.

**Methods:**

This retrospective single-center case series included 4 symptomatic male patients (mean age 68.0 years, range 59–75) with total CCA occlusion (Riles type 1A) and severe ipsilateral ICA stenosis (80%–95% by NASCET), who underwent subclavian-to-internal carotid bypass with ICA endarterectomy under cervical plexus and supraclavicular brachial plexus block with 0.5% ropivacaine between December 2021 and June 2026. Neurologic status was monitored continuously in awake patients during carotid cross-clamping. Systolic blood pressure was maintained approximately 20 mmHg above preoperative baseline to optimize collateral perfusion.

**Results:**

Primary technical success was 100%. Mean carotid cross-clamping time was 19.3 min (range, 17–21 min). Retrograde ICA stump pressure was ≥40 mmHg in all patients. No intraoperative neurologic deficits, perioperative strokes, transient ischemic attacks, cranial nerve injuries, graft thromboses, or deaths occurred. At the latest available follow-up, all grafts remained patent and no recurrent neurologic events were recorded. Twelve-month follow-up was available for Patients A, C, and D, whereas Patient B had 3 months of follow-up. One patient with contralateral 98% ICA stenosis underwent staged contralateral CEA one month later.

**Conclusion:**

In this small case series, subclavian-to-internal carotid artery bypass combined with ICA endarterectomy under regional anesthesia with awake neurologic monitoring was technically feasible and was associated with favorable observed perioperative and available follow-up outcomes. Larger comparative studies are warranted.

## Introduction

Lesions of the carotid arteries (CA) may manifest as either chronic cerebral ischemic symptoms or cerebrovascular event (1–3). Total occlusion of the common carotid artery (CCA) from its origin is a rare form of carotid pathology. According to various authors, its frequency does not exceed 1% among all stenoses of the brachiocephalic arteries (4, 5). Involvement of the entire CCA (from its origin to the bifurcation) leads to profound hemodynamic insufficiency. Collateral circulation through the Circle of Willis and the ophthalmic artery is often insufficient, especially in the presence of hypoplasia or occlusion of contralateral pathways (6–8).

The natural history of total carotid artery occlusion is characterized by a high risk of ipsilateral stroke with conservative management (9).

Surgical options for CCA occlusion include aorto-internal carotid artery bypass via a sternotomy approach (10). However, less traumatic extra-anatomic procedures are also available. These include crossover carotid-to-internal carotid bypass when the contralateral CCA is patent (11). In cases of severe contralateral CCA stenosis with a preserved ipsilateral subclavian artery (SA), subclavian-to-internal carotid artery bypass is feasible (12). Even more rarely, such operations are performed under regional anesthesia with consciousness monitoring, which represents the most informative method of intraoperative neurological monitoring (13).

**The aim** of this study was to evaluate the outcomes of subclavian-to-internal carotid artery bypass with CEA of the internal carotid artery ICA in patients with total CCA occlusion using regional anesthesia.

## Materials and methods

This study represents a retrospective clinical case series. All patients provided written informed consent for the surgical procedure, the use of their clinical data for research purposes, and the publication of their anonymized clinical data and images.

*Inclusion criteria* were total CCA occlusion with severe ipsilateral ICA stenosis, symptomatic cerebrovascular disease defined as ipsilateral ischemic stroke and/or transient ischemic attack (TIA), and treatment with subclavian-to-internal carotid artery bypass combined with ICA endarterectomy under regional anesthesia.

*Exclusion criteria* were previous carotid revascularization on the affected side, including carotid endarterectomy or carotid artery stenting, intracranial hemorrhage, and complete occlusion of the ipsilateral ICA.

### Patient characteristics

This retrospective, single-center case series included all consecutive patients who met the eligibility criteria and underwent subclavian-to-internal carotid artery bypass combined with ICA endarterectomy under regional anesthesia between December 2021 and June 2026. No eligible patients were excluded (Table 1).

**Table 1:** Clinical characteristics and follow-up of patients.

Parameter	Patient A	Patient B	Patient C	Patient D
Age (years)	59	72	75	66
Sex	male	male	male	male
Side of lesion (CCA occlusion and severe ICA stenosis)	right	left	left	left
Extent of CCA occlusion	From CCA origin to carotid bifurcation	From CCA origin to carotid bifurcation	From CCA origin to carotid bifurcation	From CCA origin to carotid bifurcation
Ipsilateral ICA stenosis (%)	80%	90%	95%	85%
Contralateral ICA stenosis (%)	40%	30%	98%	20%
History of stroke	yes	no	yes	no
Transient ischemic attack	no	yes	no	yes
Arterial hypertension	yes	yes	yes	yes
Type 2 diabetes mellitus	yes	yes	yes	yes
Coronary artery disease (CAD)	yes	yes	yes	yes
History of myocardial infarction	yes	yes	yes	yes
Follow-up available for analysis (months)	12	3	12	12

As shown in Table 1, all patients were male (100%), with a mean age of 68.0 years (range, 59–75 years). Left-sided CCA occlusion was present in three patients (75%), whereas right-sided CCA occlusion was present in one patient (25%). In all four patients, the CCA occlusion was classified as Riles type 1A (14).

All patients had symptomatic CCA occlusion. Two patients (50%) had a history of ipsilateral ischemic stroke, whereas the remaining two patients (50%) had experienced ipsilateral transient ischemic attacks (TIAs). All patients had substantial multimorbidity, including arterial hypertension (AH), type 2 diabetes mellitus (DM), coronary artery disease (CAD), and a previous myocardial infarction (MI).

Severe ipsilateral ICA stenosis was identified in all patients on the side of the CCA occlusion. According to the North American Symptomatic Carotid Endarterectomy Trial (NASCET) criteria, the degree of ICA stenosis was 80% in Patient A, 90% in Patient B, 95% in Patient C, and 85% in Patient D (15). In addition, Patient C had severe contralateral right ICA stenosis (98%), making this case particularly challenging because of the markedly increased risk of cerebral hypoperfusion during temporary clamping of the left ICA. One month after the initial procedure, the patient successfully underwent right-sided carotid endarterectomy.

### Preoperative assessment

Comprehensive preoperative vascular imaging was performed in all patients to define the anatomical characteristics of the lesion, evaluate cerebral perfusion, and plan the surgical strategy.

All patients underwent duplex ultrasound scanning (DUS) of the brachiocephalic arteries for assessment of vascular anatomy and hemodynamic parameters. The examination was performed using a linear-array transducer in grayscale, color Doppler, and spectral Doppler modes. Peak systolic and end-diastolic flow velocities were measured in the accessible carotid artery segments. Longitudinal and transverse scans were obtained to evaluate arterial anatomy, vessel wall characteristics, atherosclerotic plaque morphology, calcification, and blood-flow patterns.

Multislice computed tomography angiography (MSCTA) of the extracranial and intracranial arteries was performed using a Philips Brilliance iCT scanner with a slice thickness of up to 0.6 mm, providing high spatial resolution. Arterial and venous phase imaging after administration of a low-osmolar contrast agent enabled comprehensive evaluation of the brachiocephalic, carotid, and vertebrobasilar arteries, as well as the circle of Willis. Three-dimensional vascular reconstructions were generated using RadiAnt DICOM Viewer (version 2020.2.3), allowing precise assessment of vascular anatomy, the extent of CCA occlusion, distal arterial patency, collateral circulation, and the severity of ICA stenosis (Figures 1–3).

**Figure 1:**
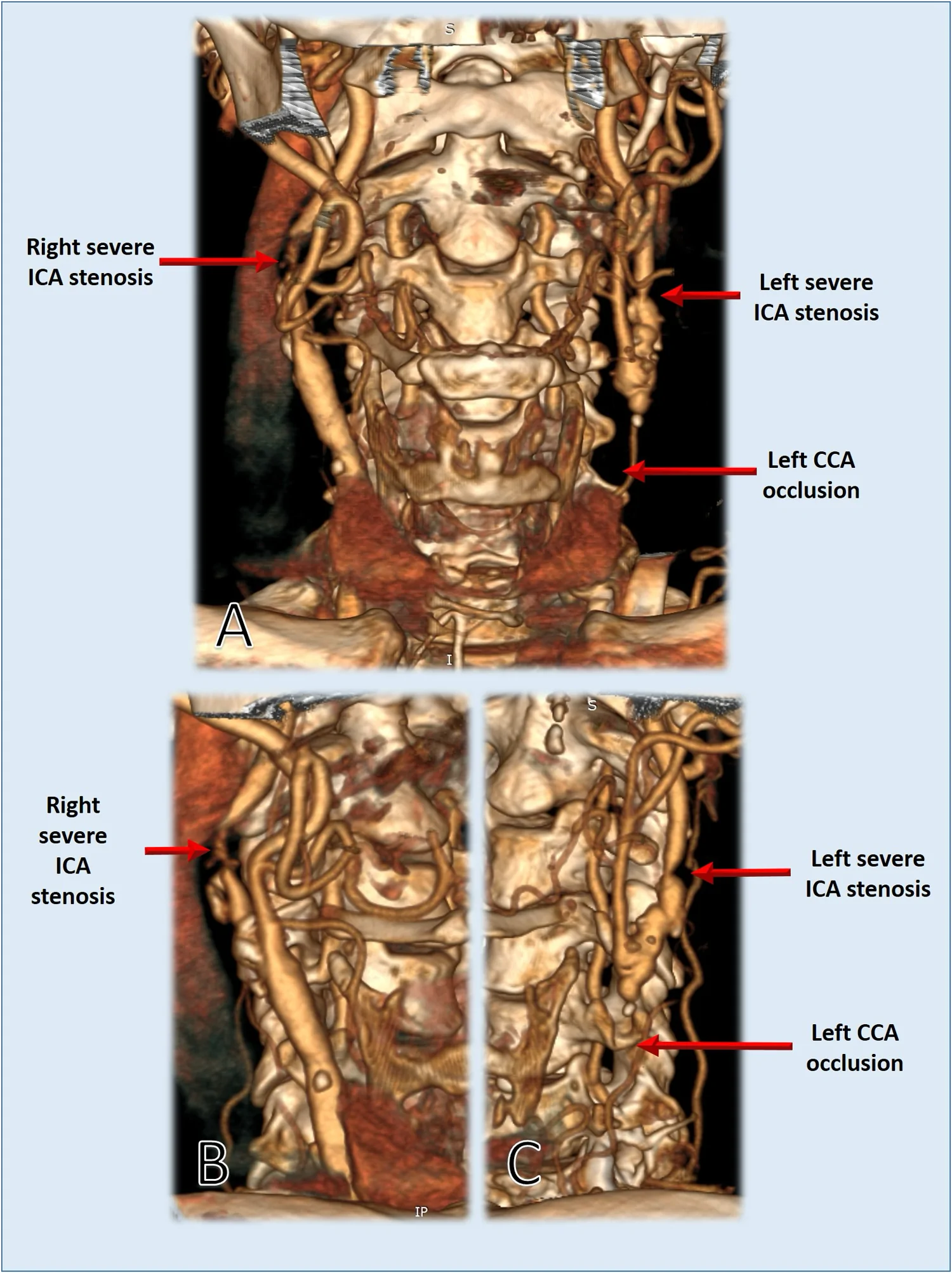
Preoperative CT angiography of extracranial and intracranial arteries with contrast enhancement (patient C). **(А)** anterior projection; **(B)** right lateral projection; **(C)** left lateral projection;.

**Figure 2:**
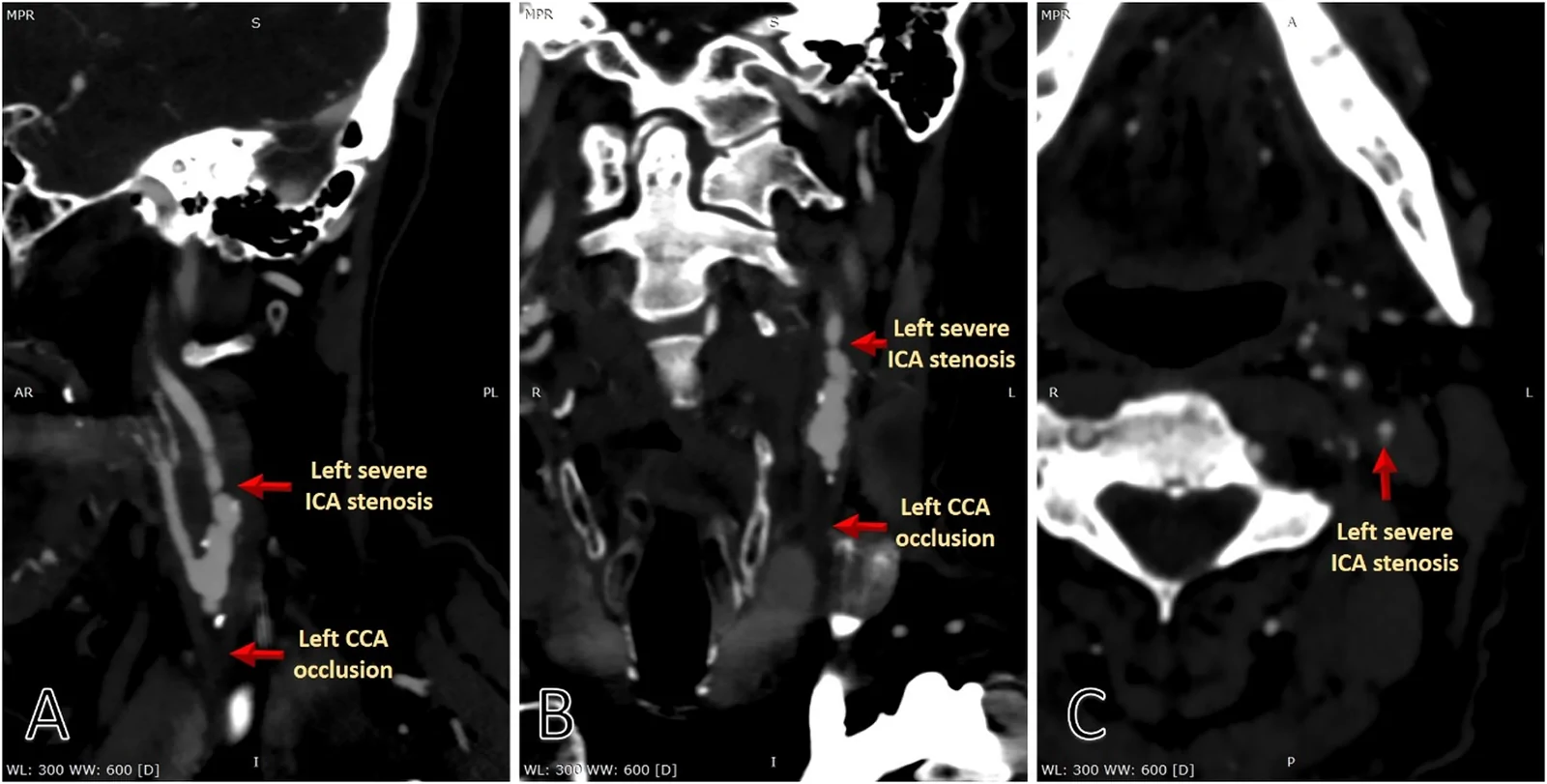
Preoperative contrast-enhanced CT angiography of patient C demonstrating severe left ICA stenosis and left CCA occlusion. **(A)** Sagittal multiplanar reconstruction; **(B)** coronal multiplanar reconstruction; **(C)** axial image.

**Figure 3:**
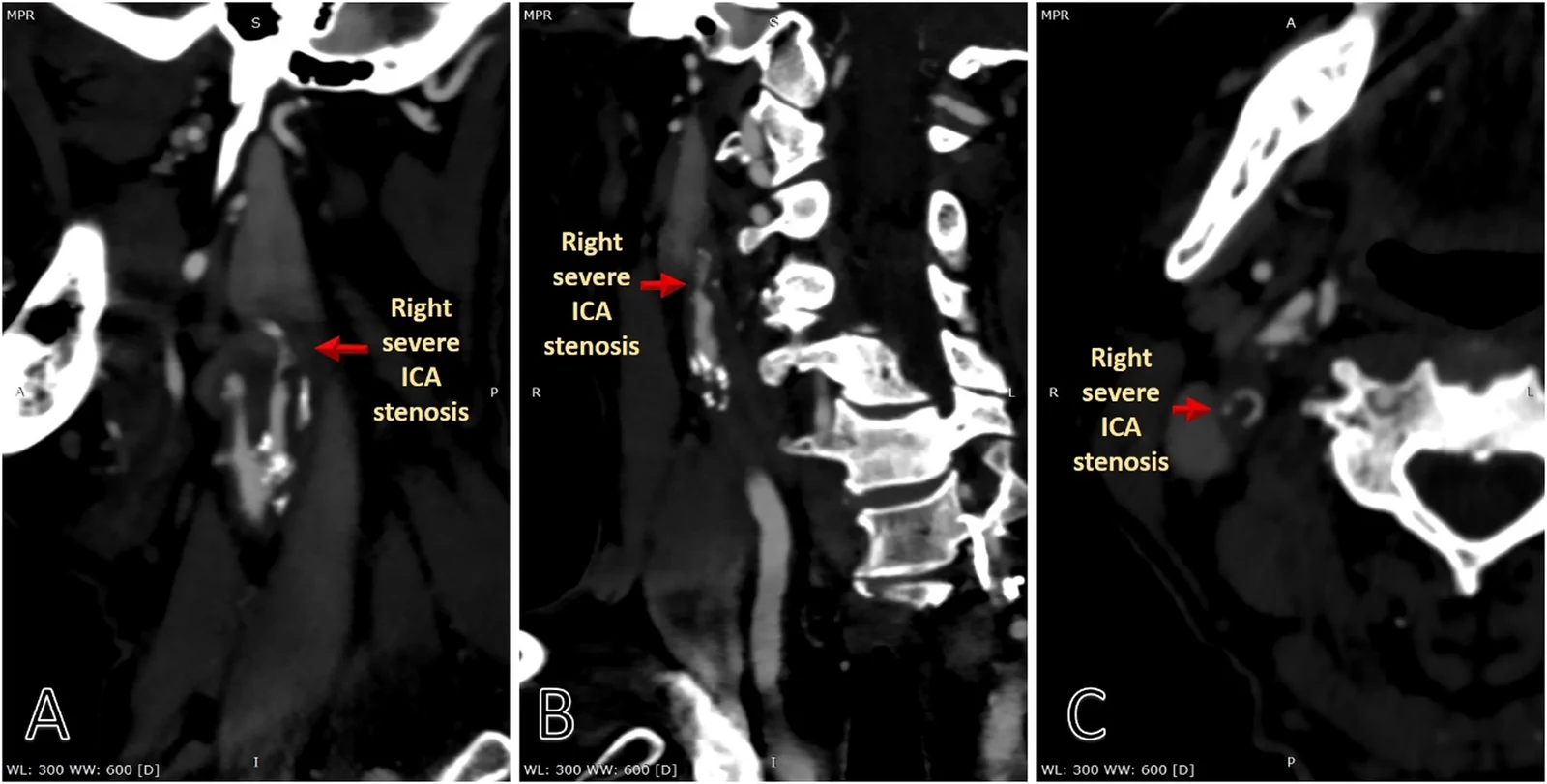
Preoperative contrast-enhanced CT angiography of patient C demonstrating severe right ICA stenosis. **(A)** Sagittal multiplanar reconstruction; **(B)** coronal multiplanar reconstruction; **(C)** axial image.

### Anesthetic management

All procedures were performed under regional anesthesia with the patients remaining fully awake throughout the operation to allow continuous neurological assessment. No intravenous sedation was administered.

All regional blocks were performed by the same experienced anesthesiologist under ultrasound guidance using a LOGIQ P7 ultrasound system (GE Ultrasound Korea, Seongnam, South Korea) equipped with a 6–13 MHz linear-array transducer. A combination of superficial and intermediate cervical plexus blocks together with a supraclavicular brachial plexus block was used. The cervical plexus blocks provided anesthesia for the carotid bifurcation and cervical surgical field, whereas the supraclavicular brachial plexus block provided anesthesia for exposure of the subclavian artery.

A 0.5% ropivacaine solution (Polpharma-Santo, Shymkent, Kazakhstan) was administered, with a total volume of approximately 15–20 mL for the combined blocks. The administered dose remained within the recommended safety limits. Before each injection, negative aspiration was performed to minimize the risk of inadvertent intravascular injection, and the local anesthetic was administered incrementally in divided, titrated doses under continuous ultrasound guidance. No additional local anesthetic infiltration of the operative field was required.

Following completion of the regional blocks, diaphragmatic excursion and respiratory function were evaluated by ultrasonography. No clinically significant diaphragmatic dysfunction or respiratory impairment was observed. Regional anesthesia provided adequate surgical conditions in all four patients, with no cases of inadequate block requiring supplemental local anesthesia. No patient required conversion to general anesthesia.

### Intraoperative monitoring

Before skin incision, baseline neurological function was assessed, including the level of consciousness, speech, orientation, motor function, and sensation.

Continuous neurological monitoring was performed throughout CA cross-clamping and after restoration of blood flow by maintaining verbal communication with the patient and repeatedly assessing speech, orientation, response to commands, hand grip strength, and voluntary movements of the upper and lower extremities. Because all procedures were performed under regional anesthesia with the patients remaining fully awake, neurological status served as the primary indicator of adequate cerebral perfusion during carotid cross-clamping.

To optimize collateral cerebral perfusion during carotid cross-clamping, systolic arterial pressure was intentionally maintained at approximately 20 mmHg above each patient's usual preoperative systolic blood pressure. In the present series, this corresponded to a maintained systolic arterial pressure of approximately 160–170 mmHg during carotid cross-clamping.

Retrograde ICA stump pressure was measured immediately after carotid clamping as an adjunctive assessment of cerebral perfusion. A retrograde ICA stump pressure of ≥40 mmHg was considered indicative of adequate collateral cerebral perfusion (Table 2). However, the decision regarding temporary intraluminal shunt placement was based primarily on continuous intraoperative neurological assessment rather than stump pressure alone. No patient developed neurological deterioration during carotid cross-clamping; therefore, temporary shunting was not required in any case.

**Table 2:** Intraoperative hemodynamic and neurological monitoring during carotid cross-clamping.

Parameter	Patient A	Patient B	Patient C	Patient D
Preoperative systolic arterial pressure (mmHg)	141	152	151	143
Maintained systolic arterial pressure during carotid cross-clamping (mmHg)	∼160	∼170	∼170	∼160
Retrograde ICA stump pressure (mmHg)	46	52	41	47

### Surgical technique

The patient was positioned supine with the head rotated to the contralateral side. The operative field was prepared three times with povidone—iodine solution. Under regional anesthesia, the common, external, and internal carotid arteries were exposed through an incision along the medial border of the sternocleidomastoid muscle. The SA was exposed through a supraclavicular approach.

Systemic anticoagulation was achieved with intravenous unfractionated heparin at an initial dose of approximately 5000 IU. The heparin dose was adjusted to achieve and maintain an activated clotting time (ACT) of 300–350 s before arterial clamping.

An arteriotomy was performed in the SA, and the proximal anastomosis was constructed in an end-to-side fashion using a 7-mm POLYTHESE® synthetic vascular graft and continuous 6-0 polypropylene (Prolene) suture. Following completion of the proximal anastomosis and restoration of flow through the graft, the graft was passed beneath the sternocleidomastoid muscle to the carotid reconstruction site.

The CCA was opened longitudinally, and the arteriotomy was extended across the carotid bifurcation onto the carotid bulb and proximal ICA, creating a T-shaped arteriotomy and providing adequate exposure for endarterectomy. Endarterectomy was performed at the carotid bifurcation and proximal ICA, followed by ligation of the proximal CCA.

The distal end of the graft was anastomosed directly to the carotid bifurcation, with the anastomosis extending onto the proximal ICA. This configuration enlarged the ICA origin while preserving blood flow to both the ICA and ECA. After completion of the reconstruction and restoration of blood flow, hemostasis was achieved, a surgical drain was placed, and the wound was closed in layers (Figures 4,5).

**Figure 4:**
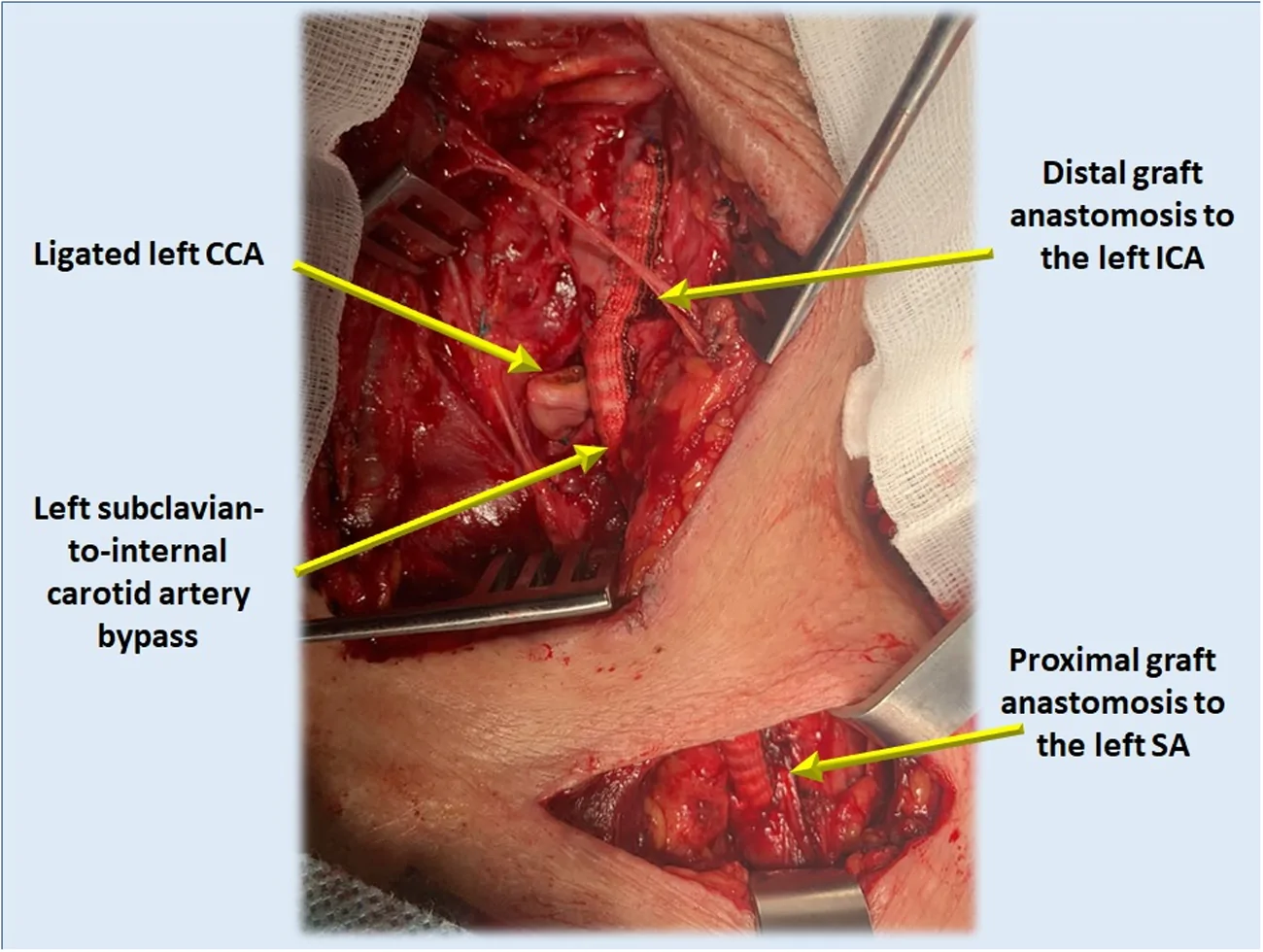
Subclavian-to-internal carotid artery bypass with carotid endarterectomy of the ICA.

**Figure 5:**
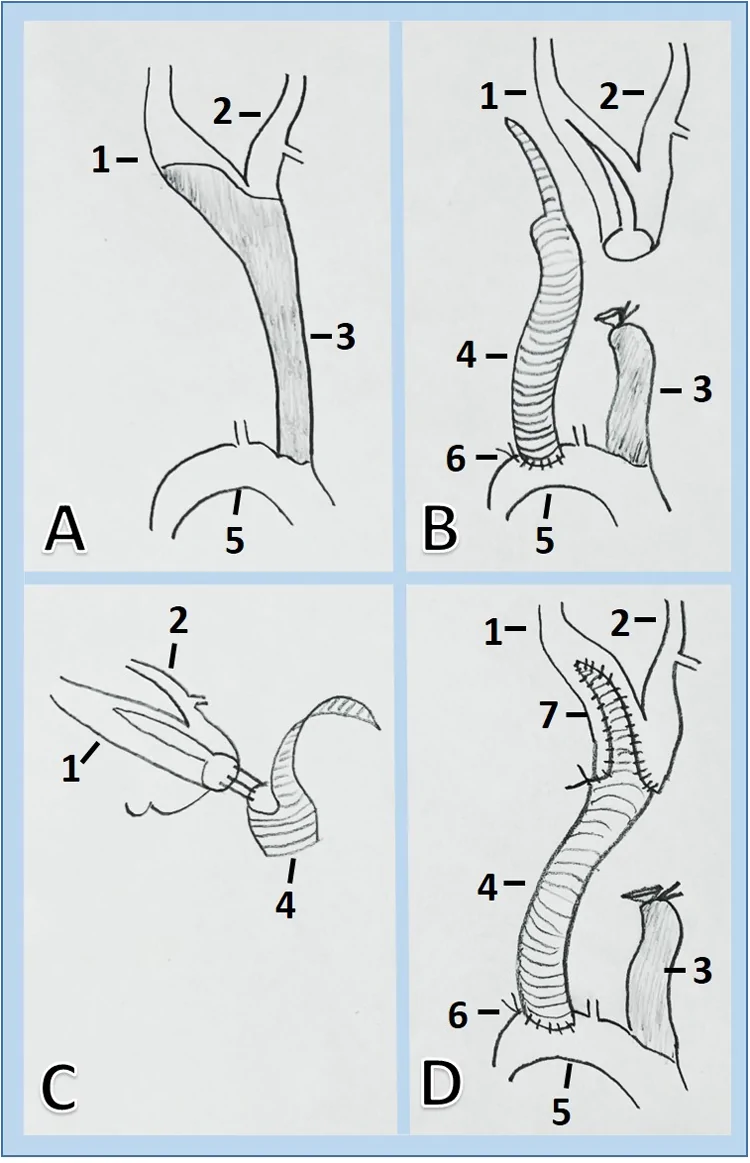
Schematic representation of subclavian-to-internal carotid artery bypass with CEA. **(A)** Preoperative anatomy demonstrating chronic total CCA occlusion from its origin to the bifurcation with severe ICA stenosis. **(B)** Construction of the proximal bypass: proximal end-to-side anastomosis of the vascular graft to the SA, with ligation and transection of the proximally occluded CCA. **(C)** CEA with construction of the distal bypass anastomosis. **(D)** Final reconstruction. 1—ICA; 2—External carotid artery (ECA); 3—CCA—ligated and transected; 4—Vascular graft; 5—SA; 6—Proximal anastomosis; 7—Distal anastomosis.

### Postoperative management

Following surgery, all patients received clopidogrel 75 mg once daily as antiplatelet monotherapy during hospitalization, which was continued after discharge as long-term therapy. Postoperatively, blood pressure was carefully monitored and maintained not below each patient's usual preoperative blood pressure to preserve adequate cerebral perfusion. Closed-suction drains were removed on the first postoperative day in all patients. The duration of hospitalization ranged from 7 to 8 days. Patients were discharged after meeting standard discharge criteria, including stable neurological status, hemodynamic stability, satisfactory wound healing without evidence of surgical complications, adequate oral intake, and independent ambulation or return to their preoperative functional status.

Patients underwent clinical and imaging follow-up at hospital discharge and during the available postoperative follow-up period. Twelve-month follow-up was available for Patients A, C, and D, whereas Patient B, who underwent surgery most recently, had 3 months of follow-up. Graft patency was assessed by duplex ultrasonography and computed tomography angiography (Figure 6).

**Figure 6:**
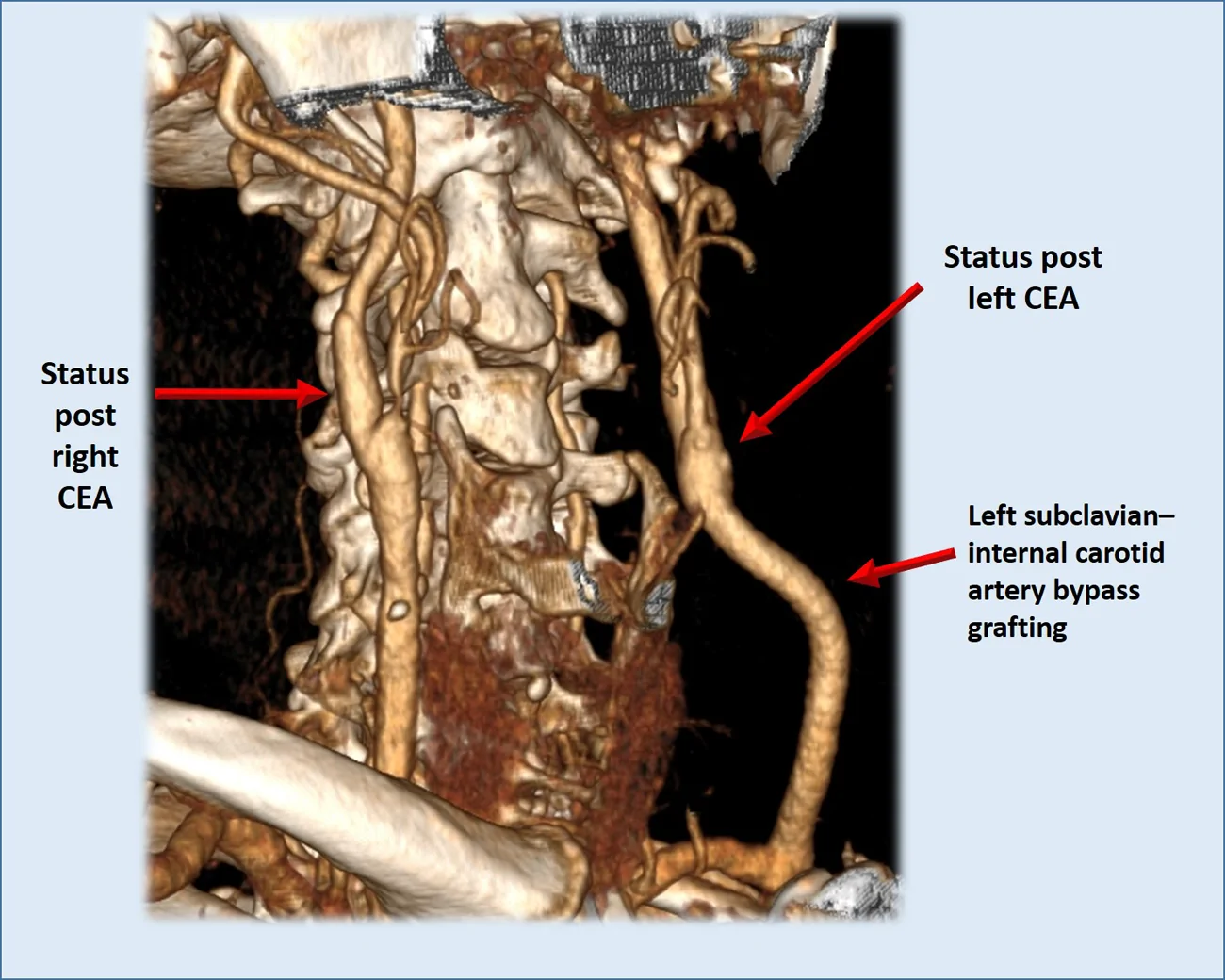
Postoperative CT angiography of extracranial and intracranial arteries with contrast enhancement (patient C).

## Results

### Intraoperative data

All 4 procedures were completed successfully according to the planned surgical technique. Primary technical success was 100%. Temporary shunting was not required in any case (Table 3).

**Table 3:** Intraoperative parameters.

Parameter	Patient A	Patient B	Patient C	Patient D
Operative time (min)	211	197	216	189
ICA clamping time (min)	20	19	21	17
SA clamping time (min)	17	15	18	16
Intraoperative blood loss (mL)	30	35	30	40

The mean ICA clamping time was 19.3 min (range 17–21 min), and the mean SA clamping time was 16.5 min (range 15–18 min). Mean intraoperative blood loss was 33.8 mL (range 30–40).

No intraoperative neurological complications, or episodes of critical retrograde pressure drop were recorded. In the early postoperative period, no bleeding, cranial nerve neuropathies, or thromboses were observed.

It should be noted that Patient C, after left-sided subclavian-to-internal carotid artery bypass with ICA endarterectomy under regional anesthesia, underwent right-sided carotid endarterectomy one month later. At 12-month follow-up, no graft thrombosis, recurrent ischemic stroke, or death was recorded in Patients A, C, and D. Patient B remained free of these events during the available 3-month follow-up.

## Discussion

The optimal surgical management of chronic CCA occlusion remains dependent on individual vascular anatomy because no single reconstructive technique is suitable for all patterns of disease. In our series, all patients had Riles type 1A occlusion associated with severe ipsilateral ICA stenosis. Under these anatomical conditions, subclavian-to-ICA bypass combined with carotid endarterectomy was selected to restore antegrade cerebral perfusion while simultaneously treating severe ipsilateral ICA stenosis. Rather than representing two independent procedures, the bypass and endarterectomy constituted complementary components of a single reconstructive strategy tailored to the underlying vascular anatomy.

Endovascular and hybrid techniques have emerged as alternative strategies for chronic carotid occlusive disease; however, their feasibility is highly dependent on lesion anatomy. In the present series, the CCA occlusion involved the vessel origin in all patients. In three patients, the left CCA was occluded from its origin at the aortic arch, whereas in one patient the right CCA was occluded from its origin at the brachiocephalic trunk. Consequently, no suitable proximal patent CCA segment was available for stable catheter engagement and guidewire support. The absence of a suitable proximal CCA stump was therefore particularly relevant to treatment selection. Previous reports have suggested that CCA occlusion located at a distance from the arterial origin may be more favorable for endovascular stenting than an ostial lesion (16). Importantly, in all four patients, the chronic CCA occlusion extended essentially throughout the entire vessel, from its origin to the carotid bifurcation, and was associated with severe tandem ipsilateral ICA stenosis. Thus, there was no patent distal CCA segment suitable for straightforward percutaneous retrograde access. A retrograde or hybrid strategy would therefore still have required surgical exposure of the carotid bifurcation, followed by retrograde traversal of the entire chronically occluded CCA up to its origin and subsequent treatment of the severe ipsilateral ICA lesion. Given the technical complexity of long-segment chronic recanalization and the potential risks of subintimal guidewire passage, vessel-wall injury, arterial dissection, and embolization, subclavian-to-ICA bypass combined with CEA was selected as the more direct and predictable reconstructive strategy. This approach avoided manipulation through the entire chronically occluded CCA while simultaneously allowing definitive treatment of the carotid bifurcation/proximal ICA lesion. Retrograde and hybrid techniques remain potential alternatives in appropriately selected patients with Riles type 1A CCA occlusion; therefore, our treatment selection should not be interpreted as implying that these approaches are universally contraindicated.

A distinguishing feature of the present series was the use of regional anesthesia with continuous awake neurologic monitoring. Unlike indirect monitoring techniques, awake assessment provides real-time evaluation of cerebral function throughout carotid cross-clamping and allows immediate recognition of neurological deterioration (17). In our practice, neurological examination represented the principal determinant of intraoperative decision-making, whereas retrograde ICA stump pressure served only as an adjunctive assessment of collateral cerebral perfusion. This strategy is consistent with current concepts of selective shunting, in which direct neurological assessment in awake patients is considered more informative than physiological surrogate markers alone. Although randomized studies have not demonstrated clear superiority of regional over general anesthesia with respect to major neurological outcomes, the ability to perform continuous neurological assessment remains an important practical advantage during complex carotid reconstruction (18). Furthermore, comparative studies suggest that regional anesthesia may be associated with lower overall perioperative morbidity compared to general anesthesia (19). The literature contains reports of carotid surgeries under regional anesthesia, however, most are limited to carotid endarterectomy alone (20–22). Our series involved more extensive reconstructive procedures, including not only endarterectomy but also vascular bypass, requiring two surgical accesses—supraclavicular and along the medial border of the sternocleidomastoid muscle.

The management of Patient C illustrates the importance of individualized operative planning in bilateral carotid disease. Severe contralateral ICA stenosis combined with complete occlusion of the opposite CCA substantially increased the anticipated risk of cerebral hypoperfusion during carotid cross-clamping. For this reason, staged reconstruction was selected, with restoration of left-sided cerebral inflow preceding right carotid endarterectomy. This sequence reduced the potential hemodynamic risk associated with the second-stage procedure and highlights the importance of adapting the operative strategy to each patient's cerebrovascular anatomy rather than applying a uniform reconstructive approach.

The present study has several limitations. First, it is a retrospective single-center case series involving only four patients, which limits the generalizability of the findings. Second, the absence of a control group precludes comparison with alternative surgical or endovascular treatment strategies. Third, follow-up was not uniform across all patients: 12-month follow-up was available for three patients, whereas the most recently treated patient had 3 months of follow-up. This limits uniform assessment of mid- and long-term graft durability and neurological outcomes. Consequently, the present findings should be regarded as descriptive and hypothesis-generating. Larger multicenter studies with longer follow-up are required to define better the role of this reconstructive strategy in the management of chronic CCA occlusion.

## Conclusion

In this four-patient case series, subclavian-to-internal carotid artery bypass combined with ICA endarterectomy under regional anesthesia was technically feasible and was associated with favorable perioperative and available follow-up outcomes. Awake neurologic assessment allowed direct monitoring during carotid clamping, and temporary shunting was not required. However, the small sample size, retrospective design, absence of a comparator group, and limited follow-up preclude conclusions regarding comparative safety or efficacy.

## Data Availability

The raw data supporting the conclusions of this article will be made available by the authors, without undue reservation.
